# Development and Validation of the Food Liking Questionnaire in a French-Canadian Population

**DOI:** 10.3390/nu9121337

**Published:** 2017-12-08

**Authors:** Elise Carbonneau, Maude Bradette-Laplante, Benoît Lamarche, Véronique Provencher, Catherine Bégin, Julie Robitaille, Sophie Desroches, Marie-Claude Vohl, Louise Corneau, Simone Lemieux

**Affiliations:** 1 Institute of Nutrition and Functional Foods, Laval University, 2440 Hochelaga Boulevard, Quebec City, QC G1V 0A6, Canada; maude.bradette-laplante.1@ulaval.ca (M.B.-L.); benoit.lamarche@fsaa.ulaval.ca (B.L.); veronique.provencher@fsaa.ulaval.ca (V.P.); julie.robitaille@fsaa.ulaval.ca (J.R.); sophie.desroches@fsaa.ulaval.ca (S.D.); marie-claude.vohl@fsaa.ulaval.ca (M.-C.V.); louise.corneau@fsaa.ulaval.ca (L.C.); simone.lemieux@fsaa.ulaval.ca (S.L.); 2 School of Psychology, Laval University, 2325 rue des Bibliothèques, Québec, QC G1V 0A6, Canada; catherine.begin@psy.ulaval.ca

**Keywords:** food liking, questionnaire, development, validation, French, Canada

## Abstract

The purpose of this study was to develop and validate a questionnaire assessing food liking in a French-Canadian population. A questionnaire was developed, in which participants were asked to rate their degree of liking of 50 food items. An expert panel evaluated the content validity. For the validation study, 150 men and women completed the questionnaire twice. An Exploratory Factor Analysis (EFA) was performed to assess the number of subscales of the questionnaire. Internal consistency and test-retest reliability of the subscales were evaluated. Concurrent validity was assessed through correlations between liking scores and self-reported frequencies of consumption. Comments from the experts led to changes in the list of foods included in the questionnaire. The EFA revealed a two-factor structure for the questionnaire (i.e., savory and sweet foods) and led to the removal of nine items, resulting in a 32-item questionnaire. The two subscales revealed good internal consistency (Cronbach alphas: 0.85 and 0.89) and test-retest reliability (*p* = 0.84 and 0.86). The questionnaire demonstrated adequate concurrent validity, with moderate correlations between food liking and self-reported frequency of consumption (*r* = 0.19–0.39, *p*s < 0.05). This new Food Liking Questionnaire assessing liking of a variety of savory and sweet foods demonstrated good psychometric properties in every validation step. This questionnaire will be useful to explore the role of food liking and its interactions with other factors in predicting eating behaviors and energy intake.

## 1. Introduction

Food choices are influenced by a variety of factors interacting with one another. When many foods are available, psychophysiological (e.g., mood, hunger), situational, and hedonic cues contribute to determine what food is wanted or desired [1,2,3]. Hedonic cues are likes and dislikes that can be innate or developed, through cultural exposition and personal experiences, among other things [2]. Being important determinants of food choices, food liking and preferences have been assessed in different ways in many studies. Existing questionnaires vary through methods of assessment. Whereas some authors have chosen to evaluate food preferences [4] (i.e., choice of a food item when two or more alternatives are presented [3]), others assessed food liking [5,6,7] (i.e., pleasantness of a food’s taste [8]), and other authors have chosen to evaluate both [9]. Questionnaires also differ with the choice of foods assessed, from wide variety of food types [5,7,10] to specific food characteristics such as fat [4] or salty, sweet, and fat [9].

In the context of a large study aiming at identifying determinants of healthy eating in the French-Canadian population of the province of Quebec, our research team wanted to create a questionnaire in order to evaluate whether a strong liking for salty, sweet, and fatty foods is a barrier to healthy eating [11,12,13]. A French questionnaire assessing liking and preferences for salt, sweet, and fat already exists [9], but it was not suitable for our needs for different reasons. The questionnaire developed by Deglaire et al. [9] is composed of four different sections, including questions on liking for different foods, preferences in the level of seasoning, preferences for types of dishes on a menu, and general questions about behaviors related to sweet, salt, and fat. Therefore, it is quite long to complete (83 items; 23 min). The questionnaire we aimed to develop was to be used as part of a comprehensive investigation comprising several other questionnaires, and in this context needed to be shorter to reduce the completion burden for the participants. Also, as recommended by Beaton et al. [14], a cultural adaptation is essential when a tool is to be used in different countries, even if the same language is spoken. In the case of a food liking questionnaire, it is all the more important to adapt the list of foods for the targeted population. The questionnaire developed by Deglaire et al. [9] was specific to France and hence, the list of food items was not applicable to French-speaking populations in Canada. Therefore, the purpose of this study was to develop and validate a food liking questionnaire adapted to an adult French-Canadian population.

## 2. Materials and Methods

### 2.1. Development of the Questionnaire

We decided to develop the Food Liking Questionnaire in order to evaluate whether a strong liking of salty, sweet, and fatty foods is a barrier to healthy eating. Therefore, only foods either rich in salt, sugar, or fat were included in the questionnaire. In order to develop a complete list, we first opted to classify such foods into four main categories: high-salt/high-fat, high-salt/low-fat, high-sugar/high-fat, and high-sugar/low-fat. However, as observed by other authors [9], very few food items belong to the high-salt/low-fat category; therefore, we decided not to include this category. The list of food items was developed based on existing questionnaires in the literature [4,9,15], and a food frequency questionnaire that was developed especially for the French-Canadian population [16]. In order to be able to assess the liking for sweet and salty foods precisely, we identified foods that stood on their own (e.g., salted nuts, French fries, cookies), unlike other authors [4,9] who assessed combinations of food items (e.g., cream cheese on a bagel, potatoes with butter, fruits served with whipped cream). These combinations may create confusion to participants who do not equally like the two food items grouped together (e.g., strong liking for bagels, but low liking of cream cheese). A 50-item list was developed (19 high-salt/high-fat foods, 16 high-sugar/high-fat foods, and 15 high-sugar/low-fat foods). Respondents are asked to rate their liking for each food item on a nine-point scale, 1 being “I really don’t like” and 9 being “I really like”, based on what was previously done in other studies [5,7,9,10]. The “I have never tasted this food” option was also proposed. In order to develop a relatively short questionnaire, it was decided that only the liking (i.e., pleasantness of a food’s taste) would be assessed, in contrast with other questionnaires also assessing food preferences (i.e., choice of a food item when two or more alternatives are presented). 

### 2.2. Participants and Procedures

The Food Liking Questionnaire and four other questionnaires on determinants of healthy eating [17,18,19] were developed and validated in the context of a large validation research project. Three steps were followed in the validation process of the Food Liking Questionnaire, namely an expert panel evaluation, a pretest, and a validation study.

#### 2.2.1. Expert Panel

A group of four nutrition researchers, one psychology researcher, and one registered dietitian formed the expert panel that assessed the content validity by reviewing and commenting on the questionnaire and on the list of food items. The registered dietitian is also a research assistant and has an expertise in administrating and codifying food frequency questionnaires and food recalls.

#### 2.2.2. Pretest

A sample of 31 men and women from the Québec City metropolitan area were recruited for the assessment of face validity. Recruitment was performed through an internal list of people willing to participate in clinical studies. Participants had to be aged between 18 and 65 years and to have at least minimal informatics abilities to be able to complete the series of questionnaires online. The pretest was conducted according to the guidelines laid down in the Declaration of Helsinki and received approval from the Research Ethics Committee at Laval University (#2014-128/02-07-2014). Implicit informed consent was obtained from all participants.

Face validity is a validation step aiming at assessing the acceptability and comprehension of the items. Participants from the pretest were thus asked to complete the questionnaire online and to comment on the ambiguity of the items. Through participants’ comments, the developers can evaluate whether the questionnaire seems to measure what it is supposed to measure [20].

#### 2.2.3. Validation Study

A total of 150 men and women took part to the validation study. They were recruited through electronic mailing lists comprising Laval University students and employees, as well as people interested in participating in studies at the Institute of Nutrition and Functional Foods. As for the pretest, they had to be aged between 18 and 65 years, and to have at least minimal informatics skills. Pregnant and lactating women were not included. Participants also had to be free from any condition affecting intestinal absorption, since blood biomarkers of fruit and vegetable intake were also being validated in the context of the study. 

In the validation study, participants first came to the research center for a blood sample and anthropometric measurements. Height was measured to the nearest millimeter, and body weight was measured to the nearest 0.1 kg on a calibrated balance. Measures were taken by trained professionals according to standardized procedures [21]. After their visit to the research center, participants were asked to complete, at home, the series of questionnaires to be validated on the Internet platform of the study. They also had to complete a sociodemographic questionnaire and a web-based food frequency questionnaire (FFQ) validated in a French-speaking Canadian population [16]. Participants were asked to complete all questionnaires, in random order, within 30 days. After a two-week period, participants were asked to complete the questionnaires a second time (except for the sociodemographic questionnaire and the FFQ), within another 30-day window.

Participants were given a CAN$50 financial compensation for their participation in the study. This study was conducted according to the guidelines laid down in the Declaration of Helsinki. The validation study received approval from the Research Ethics Committee at Laval University (#2014-128/02-07-2014) and all participants gave written informed consent. 

### 2.3. Statistical Analyses

The analyses described in this section were performed on data derived from the validation study. An Exploratory Factor Analysis (EFA) was performed using data from the first completion to uncover the underlying structure of the Food Liking Questionnaire. The procedure aims at retaining as few factors (or subscales) as possible while explaining most of the variation in the data. The most commonly used methods to identify the adequate number of factors are the eigenvalue-greater-than-one rule (modified version of the rule proposed by Larsen and Warne [22]), the scree plot [23], and the analysis of the variance explained [24]. Internal consistency reliability was assessed for each subscale using Cronbach alpha coefficients using data from the first completion of the questionnaire. Intra-class correlation analyses were conducted between scores of the two completions of the Food Liking Questionnaire to assess test-retest reliability. Concurrent validity is a measure of how well a particular test correlates with a presumably related construct. In the present study, concurrent validity was assessed with Pearson’s correlation analyses between liking scores of each item and self-reported frequencies of consumption of equivalent foods (derived from the FFQ). Concurrent validity was not assessed for items that had no equivalent in the FFQ. Statistical tests were two-sided and differences or associations at *p* < 0.05 were considered significant. Analyses were performed using the Statistical Analysis Software (SAS) version 9.4 (Copyright© 2013, SAS Institute Inc., Cary, NC, USA).

## 3. Results and Discussion

### 3.1. Expert Panel

#### Content Validity

Following suggestions by the experts, a “neutral” label was added in the middle of the nine-point scale. Therefore, three labels appeared on the scale: (1) I really don’t like; (5) Neutral; and (9) I really like. One food item was added to the scale and seven items were judged not to be common enough to be part of the scale and were thus removed. Finally, food items that were considered to be similar were combined into a single item (e.g., various types of cheese were grouped under the “cheese” item). As a result, the questionnaire comprised 41 food items.

### 3.2. Pretest

A total of 17 women and 14 men participated in the pretest (mean age: 45.6 ± 13.9 years; range: 23–66 years).

#### Face Validity

Four participants did not complete the Food Liking Questionnaire, therefore 27 participants were included for face validity. Many participants mentioned liking a food without eating it frequently. According to comments received, it also appeared that some of them had rated their frequency of consumption rather than their actual liking of the food. Therefore, the questionnaire instructions were modified from “rate your liking of the following foods” to “rate your liking of the following foods, regardless of the frequency of consumption”. Face validity did not lead to any other change.

### 3.3. Validation Study

Of the 150 participants in the validation study, one participant dropped out of the study before completing all questionnaires. Six participants had more than 10% of missing data in the Food Liking Questionnaire and were therefore excluded from the analysis. Characteristics of the 143 participants included in the validation study are presented in Table 1. The mean (±SD) completion time of the questionnaire was 5.4 ± 3.2 min. The short time required for the questionnaire completion, compared to other existing tools (e.g., 23 min for the questionnaire by Deglaire et al. [9]), is adequate, as we developed this questionnaire for a study where participants will be asked to complete several questionnaires.

#### 3.3.1. Exploratory Factor Analysis

The EFA was performed on the 41 questionnaire items. The significance of Bartlett’s test of sphericity (khi^2^ = 2056.22, *p* < 0.0001) and the Kaiser-Meyer-Olkin test of sampling adequacy (measure of sample adequacy = 0.73) justified the use of an EFA given the common variance of the set of items [25]. According to the modified eigenvalue-greater-than-one rule [22], 15 factors could be considered to explain the variance in the data. However, the analysis of the scree plot from the EFA revealed a notable difference in the slope after the first two factors. Moreover, only two factors accounted for more than 10% of the variance in the data [24], more precisely accounting for 36.79% and 12.60% of the variance. Therefore, it was decided that the items of the questionnaire would be divided into two main factors. In order to decide between an orthogonal and an oblique rotation, an oblique (promax) rotation was first requested to obtain a correlation matrix, as recommended by Tabachnick and Fidell [25]. The correlation between the two factors exceeded 0.32, suggesting that the overlap in variance among factors justified the use of an oblique rotation [25]. Using a minimum loading cut-off of 0.30 or higher, 12 items were found to load on the first factor and 20 on the second factor (see Table 2). A total of nine items were removed from the questionnaire, seven due to low factor loadings (<0.30) and two due to cross-loading (i.e., loading ≥ 0.30 on both factors). The first factor comprised only savory foods and the second factor included high-sugar/high-fat and high-sugar/low-fat foods (from now on, the two factors will be considered as the savory subscale and the sweet subscale). The 32 food items included in the questionnaire are presented in Table 2. 

Since the food item list was elaborated based on three categories (high-salt/high-fat, high-sugar/high-fat, and high-sugar/low-fat foods), it was expected that a three-factor structure would be uncovered by the EFA. The actual structure regrouping all the sweet items in one factor, regardless of the fat content, suggests that the sweet taste prevails on the fat taste for food liking categories. 

Out of the nine items removed from the questionnaire, six sweet foods were removed due to low loading or cross-loading. These items (e.g., fruit juice, dried fruits, and yogurt) were less sweet or related less to dessert than the rest of the list, which could explain that they did not fit with the sweet factor. Butter and cheese were among the savory foods that were removed from the questionnaire. These foods may be less likely to be eaten alone in contrast to the other foods in the questionnaire, and this may explain their low loading on the savory factor. 

Since the variance explained by the two factors (49.4%) was lower than the cut-off (70%) suggested by O’Rourke and Hatcher [24], we tested the questionnaire structure with three or four factors instead of two, to evaluate whether this would increase the variance explained by the model. However, these tests did not lead to any interpretable structure. Therefore, we decided that the original two-factor structure described previously was the most adequate for our questionnaire. 

Mean scores for the two subscales are presented in Table 3. As was observed in other studies [26,27], men had significantly higher scores than women for the savory subscale (*p* = 0.0057; see Table 3). Scores were, however, similar for the sweet subscale (*p* = 0.73; see Table 3), unlike results obtained in other studies where men had higher liking for sweet foods [27], and women had higher fat-and-sweet liking [26]. In the present study, liking scores were significantly lower in participants aged 50 to 65 years compared to younger participants (*p*s = 0.0005 for savory and sweet liking; see Table 3). The same pattern of differences according to age were obtained when analyzing men and women separately (data not shown). Similarly, results from Lampure et al. [26,27] suggest that older participants are less likely to have strong sweet, fat-and-sweet, and fat-and-salt liking than younger participants. These results are consistent with studies suggesting that age is also associated with healthier food choices, which can result from lower liking for sweet and savory foods [28,29,30,31]. In the present sample, liking scores were not significantly different between body mass index (BMI) categories (*p* = 0.63 and *p* = 0.87, respectively, for savory and sweet liking; see Table 3). When evaluating differences between BMI categories in men and women separately, the results remained the same. The relation between adiposity and preferences or taste perception is subject to heterogeneous results in the literature. Some studies suggest that obese and overweight participants are more likely to have higher liking for sweet, savory, or fatty foods than normal-weight participants [26,32,33], whereas others have found no relation between sweet preference and adiposity [6,34,35].

#### 3.3.2. Internal Consistency Reliability

As seen in Table 2, both subscales were considered internally reliable, with Cronbach alpha coefficients being >0.70.

#### 3.3.3. Test-Retest Reliability

A total of 139 participants completed the questionnaire twice. The mean (±SD) lapse between test and retest was 40.9 ± 11.5 days. As shown in Table 2, intra-class correlation coefficients between repeated measures are considered good (i.e., between 0.75 and 0.90) [36] for both subscales. The high correlation coefficients observed in the present paper and in previous studies [5,6,7,8] suggest that food liking is stable over time, which is in line with the biological predispositions, the personal and cultural experiences, and the learning processes involved in the development of individual food liking and preferences [2].

#### 3.3.4. Concurrent Validity

Ten out of 12 items included in the savory subscale and 10 out of 20 items included in the sweet subscale had a matching food item in the FFQ. Moderate correlations between liking and eating frequency were observed for all items (*r* ranging from 0.19 to 0.39, *p*s < 0.05; see Table 2), suggesting a good concurrent validity. The fact that liking and eating frequency were not strongly correlated may be due to the fact that participants were able to dissociate their food liking from their habitual food consumption. These results also support the fact that food liking only explains a part of the variation in food choices and eating behaviors [1].

### 3.4. Strengths and Limitations

The three-step validation process of the questionnaire constitutes a major strength of this study. It was expressly designed for questionnaire validation, resulting in a rigorous process. However, external validity is limited due to the mostly Caucasian and highly educated sample that is not fully representative of the whole French-speaking Canadian population [37]. Another limitation of this study is the fact that the questionnaire we validated assesses recalled food liking, and was not tested jointly with actual food tasting. The questionnaire could benefit from further construct validation with participants also reporting their degree of liking of foods actually tasted in a laboratory. Such validation should also include measures of hunger/satiety and food reward, as these variables can interact with food liking in predicting eating behaviors and energy intake [8].

## 4. Conclusions

The present study aimed at developing and validating the Food Liking Questionnaire for use in French-Canadian populations. The questionnaire contains 32 items assessing liking for savory and sweet foods and demonstrated good psychometric properties in every validation step. Food liking and preferences are known to contribute to food choices [1]. This questionnaire will be useful to explore the role of food liking and its interactions with other factors in predicting healthy dietary habits, eating behaviors, and energy intake, and to assess to what extent liking of sweet or savory foods constitutes a barrier toward adherence to healthy eating recommendations. The questionnaire developed in this study is a valid and reliable tool for the French-speaking Canadian adult population, but additional validation has to be performed if the tool is to be used in other populations. 

## Figures and Tables

**Table 1:** Sample characteristics of the validation study (*n* = 143).

Characteristic	% (*n*) or M ± SD
Female	49.7 (71)
Age (years)	47.4 ± 13.3
18–34 years	25.2 (36)
35–49 years	20.3 (29)
50–65 years	54.5 (78)
Ethnicity	
Caucasian	95.8 (137)
Highest level of education	
High school	9.8 (14)
College	30.1 (43)
University	60.1 (86)
Occupation	
Worker	69.9 (100)
Retired	20.3 (29)
Student	6.3 (9)
No job	1.4 (2)
Prefer not to answer	2.1 (3)
Body mass index (kg/m^2^)	25.6 ± 4.4
Normal weight	48.2 (69)
Overweight	37.8 (54)
Obese	14.0 (20)

**Table 2:** Subscales’ items, factor loadings, and results from the concurrent validity, internal consistency reliability, and test-retest reliability.

Subscale	Items	Factor Loadings	Correlation with Self-Reported Consumption	Cronbach α	Test-Retest *p* (95% CI)
Savory	Chicken wings	0.57	0.37 **	0.85	0.86 (0.81–0.90)
	Salted nuts	0.42	†		
	Meat pâté (*creton*)	0.50	†		
	Nuggets	0.57	0.28 *		
	Chips	0.43	0.36 **		
	French fries	0.36	0.22 *		
	Hamburger	0.64	0.22 *		
	Hotdogs	0.73	0.31 *		
	Pizza	0.33	0.23 *		
	Poutine	0.52	0.39 **		
	Sausages	0.55	0.21 *		
	Processed meat	0.60	0.33 **		
Sweet	Popsicles	0.58	†	0.89	0.84 (0.70–0.88)
	Candies	0.54	0.23 *		
	Jam	0.55	†		
	Marshmallow	0.63	†		
	Honey	0.41	†		
	Pudding	0.74	†		
	Fruit salad	0.38	0.20 *		
	Sorbet	0.45	†		
	Doughnut	0.52	0.24 *		
	Cookies	0.63	0.19 *		
	Pastries (*chausson*, *chocolatine*)	0.76	†		
	Milk chocolate/chocolate bar	0.44	0.31 *		
	Cream puff/chocolate *éclair*	0.60	0.20 *		
	Whipped cream	0.53	0.20 *		
	Ice cream	0.60	0.23 *		
	Cheese cake	0.60	†		
	Muffin	0.39	0.29 *		
	Sugar fudge	0.50	†		
	Pie	0.64	0.26 *		
	Chocolate spread	0.44	†		

Note: ** *p* < 0.0001; * *p* < 0.05; † No matching item in the Food Frequency Questionnaire (FFQ); CI: confidence interval.

**Table 3:** Mean liking scores for the total sample, and according to gender, age, and body mass index (BMI).

	Savory	Sweet
Total	6.30 ± 1.24	5.60 ± 1.32
Sex		
Women	5.94 ± 1.27	5.47 ± 1.38
Men	6.66 * ± 1.10	5.73 ± 1.25
Age		
18–34 years	6.87 ± 0.97	6.20 ± 1.28
35–49 years	6.66 ± 1.05	5.85 ± 1.20
50–65 years	5.90 ** ± 1.28	5.23 ** ± 1.26
BMI		
Normal weight	6.21 ± 1.32	5.65 ± 1.40
Overweight	6.31 ± 1.18	5.49 ± 1.28
Obese	6.55 ± 1.11	5.74 ± 1.16

Note: Score range: 1 to 9 points. * Savory score significantly higher in men than women (*p* = 0.0057). ** Savory and sweet scores significantly lower among participants aged 50 to 65 years than younger participants (*p*s = 0.0005).
